# Blood pressure lowering in acute intracerebral hemorrhage

**DOI:** 10.18632/aging.101637

**Published:** 2018-11-07

**Authors:** Olalla Pancorbo, David Rodriguez-Luna

**Affiliations:** 1 Stroke Unit, Department of Neurology, Vall d’Hebron University Hospital, Barcelona, Spain; 2 Stroke Research Group, Vall d’Hebron Research Institute, Barcelona, Spain; 3 Department of Medicine, Autonomous University of Barcelona, Barcelona, Spain

**Keywords:** intracerebral hemorrhage, blood pressure, outcome

Intracerebral hemorrhage (ICH) accounts for about 10% of strokes and is associated with higher morbidity and mortality rates than other stroke subtypes. Despite its devastating effects and social burden, no proven treatment has been consistently demonstrated to be effective in ameliorating ICH consequences [1].

Elevation in blood pressure (BP) is common in acute ICH and is associated with greater hematoma expansion and poor clinical outcomes [2]. That is why BP is considered a major target in the acute period of ICH [1]. However, although safe, the relationship between intensive BP reduction and improvement of outcomes has not been consistently demonstrated in large randomized controlled trials [3–5].

The Intensive Blood Pressure Reduction in Acute Cerebral Hemorrhage Trial (INTERACT) showed a trend towards lower hematoma expansion at 24 hours in patients under an intensive BP management regimen (target systolic BP of <140 mm Hg) [3]. Despite the promising pilot results of INTERACT, INTERACT2 failed to show a significant reduction in the rate of death or major disability with intensive BP lowering at 90 days [4]. However, intensive BP lowering showed improvement on functional outcomes in an ordinal analysis of modified Rankin scale scores. Thus, INTERACT2 resulted in revisions of guidelines for the management of acute ICH, supporting systolic BP lowering to <140 mm Hg [1]. The more recent Antihypertensive Treatment of Acute Cerebral Hemorrhage II (ATACH-II) trial has not, however, corroborated INTERACT2 results [5].

Although both INTERACT and ATACH-II trials showed a trend to lower frequency of significant hematoma expansion in patients under intensive BP management [4,5], several limitations in both trials and in INTERACT2 may have resulted in failing to demonstrate a clear overall effect on hematoma expansion. First, small hematomas observed in these trials are not prone to present hematoma expansion and could have influenced that significant hematoma expansion rate in even the conservatively treated patients (15% in INTERACT, around 25% in INTERACT2 and ATACH-II) was lower than the one third seen in observational studies [2–5]. Second, hematoma expansion analyses were restricted to those patients with repeat computed tomography at 24 hours (only a third in INTERACT2), which may have biased selection towards those patients with better clinical status at baseline [4]. Third, because many patients in non-intensive groups received therapy to lower BP in these trials, the results almost certainly underestimated the effects of intensive therapy on hematoma expansion. And fourth, a long time to achieve BP control. Antihypertensive treatment was started at a median of 4 hours from symptoms onset in INTERACT-2, and only a third of the intensive treatment group achieved the target systolic BP within the first hour of treatment [4]. Similarly, although ATACH-II reported BP control in nearly 90% of patients, it was achieved 2.5 hours after antihypertensive treatment initiation [5]. Because hematoma expansion occurs in the first few hours after ICH onset, it is possible that this delay to achieve BP target could have mitigated any effect on outcomes.

A meta-analysis of five randomized controlled trials revealed greater reduction of hematoma expansion with intensive BP management, supporting the hypothesis that intensive BP reduction attenuates hematoma expansion [6]. Additional data from a secondary analysis of INTERACT2 suggest that patents with the least hematoma expansion are those who achieve target systolic BP of <140 mm Hg within the first hour [7]. Thus, a key issue of therapeutic benefit of intensive BP reduction on hematoma expansion is likely to be time. A recent study showing that high prehospital systolic BP is related to larger ICH volume on admission reinforces this hypothesis [8]. These findings suggest that early and rapid BP lowering can be crucial to attenuate ICH expansion. Furthermore, studies have shown that other BP variables such BP variability are related to hematoma expansion and subsequent poor outcome, suggesting that not only is important to keep BP low but also to maintain it stable during the acute phase of ICH [2].

To achieve early and rapid BP lowering, it would be essential to design interventions to improve time metrics in patients with acute ICH, including early ICH diagnosis and BP lowering treatment initiation, as well as rapid BP target achievement using intravenous antihypertensive agents as soon as possible. Recent approaches include BP-lowering initiation by emergency medical services in two different scenarios: conventional ambulances and mobile stroke units. In conventional ambulances, it is possible to start BP lowering before hospital admission with oral or transdermal agents by paramedics with an initial target systolic BP of <180 mm Hg in all patients with presumed stroke in a thrombolysis window, and continue BP lowering to <140 mm Hg after in-hospital ICH diagnosis. Conversely, diagnosis of ICH in mobile stroke units would allow not only to target systolic BP of <140 mm Hg in the prehospital setting, but also early administration of intravenous antihypertensive agents to allow a rapid BP lowering [8].

In our opinion, additional trials where patients with acute ICH were randomized to early, rapid, intensive, and maintained BP lowering are warranted. Such data could confirm the hypothesis that BP lowering attenuates hematoma expansion and improves clinical outcomes in patients with acute ICH.
